# Male Hypogonadism and Disorders of Sex Development

**DOI:** 10.3389/fendo.2020.00211

**Published:** 2020-04-15

**Authors:** Romina P. Grinspon, Ignacio Bergadá, Rodolfo A. Rey

**Affiliations:** ^1^Centro de Investigaciones Endocrinológicas “Dr. César Bergadá” (CEDIE), CONICET—FEI—División de Endocrinología, Hospital de Niños Ricardo Gutiérrez, Buenos Aires, Argentina; ^2^Departamento de Biología Celular, Histología, Embriología y Genética, Facultad de Medicina, Universidad de Buenos Aires, Buenos Aires, Argentina

**Keywords:** AMH, DSD, gonadal dysgenesis, steroidogenesis, testosterone

## Abstract

Disorders of Sex Development (DSD) are congenital anomalies in which there is a discordance between chromosomal, genetic, gonadal, and/or internal/external genital sex. In XY individuals, the process of fetal sex differentiation can be disrupted at the stage of gonadal differentiation, resulting in gonadal dysgenesis, a form of early fetal-onset primary hypogonadism characterized by insufficient androgen and anti-Müllerian hormone (AMH) production, which leads to the development of ambiguous or female genitalia. The process of sex differentiation can also be disrupted at the stage of genital differentiation, due to isolated defects in androgen or AMH secretion, but not both. These are forms of fetal-onset hypogonadism with dissociated gonadal dysfunction. In this review, we present a perspective on impaired testicular endocrine function, i.e., fetal-onset male hypogonadism, resulting in incomplete virilization at birth.

## Introduction to Disorders of Sex Development and Hypogonadism

Disorders of Sex Development (DSD) are congenital anomalies in which there is a discordance between chromosomal, genetic, gonadal, and/or internal/external genital sex, as defined by the Chicago consensus published in 2006 (1), more recently revised and endorsed by all major societies of pediatric endocrinology (2). The etiologies and related pathogeneses of DSD can be comprehended once the developmental physiology of fetal sex differentiation is understood, as briefly described below. Most DSD affect the fetal endocrine and/or paracrine hormonal action resulting in the wide spectrum of clinical and hormonal phenotype of these patients.

Hypogonadism is usually defined as the gonadal failure resulting in an impaired steroid hormone production and deficient gamete output. Although steroids and gametes have been recognized as gonadal products for over a century, in the last decades of the 20th century it became evident that the somatic component of the ovaries and testes also secrete protein hormones, like inhibins and anti-Müllerian hormone (AMH), which have a relevant role as biomarkers in reproductive physiology from fetal life through adulthood (3, 4). Therefore, in face of the changes occurring in the physiology of the hypothalamic-pituitary gonadal axis from fetal life to puberty, the definition of hypogonadism should be extended to the impaired function of the ovaries or testes, as compared to what is expected for age, that involves a decreased function of the germ and/or somatic (Sertoli/granulosa, Leydig/theca) cell populations of the gonads, which can result in impaired hormone secretion (estrogens, progestins, androgens, inhibins, and/or AMH) and/or gamete production (4–6).

### Physiology of Fetal Sex Differentiation

**Figure 1:**
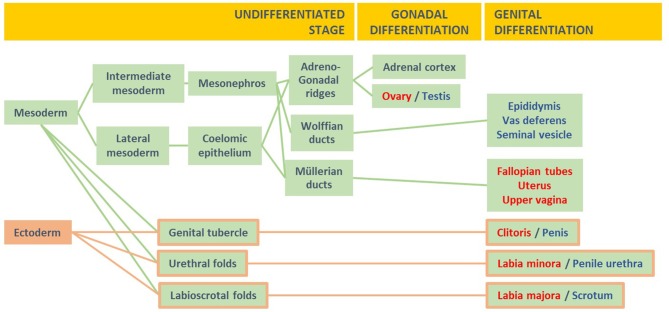
The three stages of fetal sex differentiation. During the undifferentiated stage, the mesoderm gives rise to the adreno-gonadal primordia and the Wolffian and Müllerian ducts, whereas the ectoderm and mesoderm contribute to the formation of the genital tubercle and the urethral and labioscrotal folds. During the stage of gonadal differentiation, the testes or ovaries develop from the gonadal ridges. During the stage of genital differentiation, the internal and external genitalia differentiate along the male or the female pathway. Modified with permission from Rey et al. (7) © 2018 McGraw Hill Education.

The process of sexual differentiation in fetal life can be divided into three stages (Figure 1): (i) the formation of the undifferentiated gonads and the anlagen of the genitalia, internal (Müllerian and Wolffian ducts, urogenital sinus) and external (genital tubercle, urethral folds, labioscrotal folds), (ii) gonadal differentiation, and (iii) male or female differentiation of the internal and external genitalia (8).

The initial formation of the undifferentiated gonads, the Müllerian and Wolffian ducts, the urogenital sinus and the undifferentiated external genitalia is identical in the XX and the XY fetuses between the 3rd and the 5th weeks of embryonic life (i.e., 5–7 weeks of amenorrhea). The gonadal ridges, the urogenital sinus, and the external genitalia are bipotential, i.e., they can develop through either the female or the male pathways. The internal ducts are unipotential: Müllerian ducts can give rise only to female functional structures (the Fallopian tubes, uterus, and upper vagina), and Wolffian ducts can differentiate only into male structures (the epididymides, *vasa deferentia*, and seminal vesicles).

**Figure 2:**
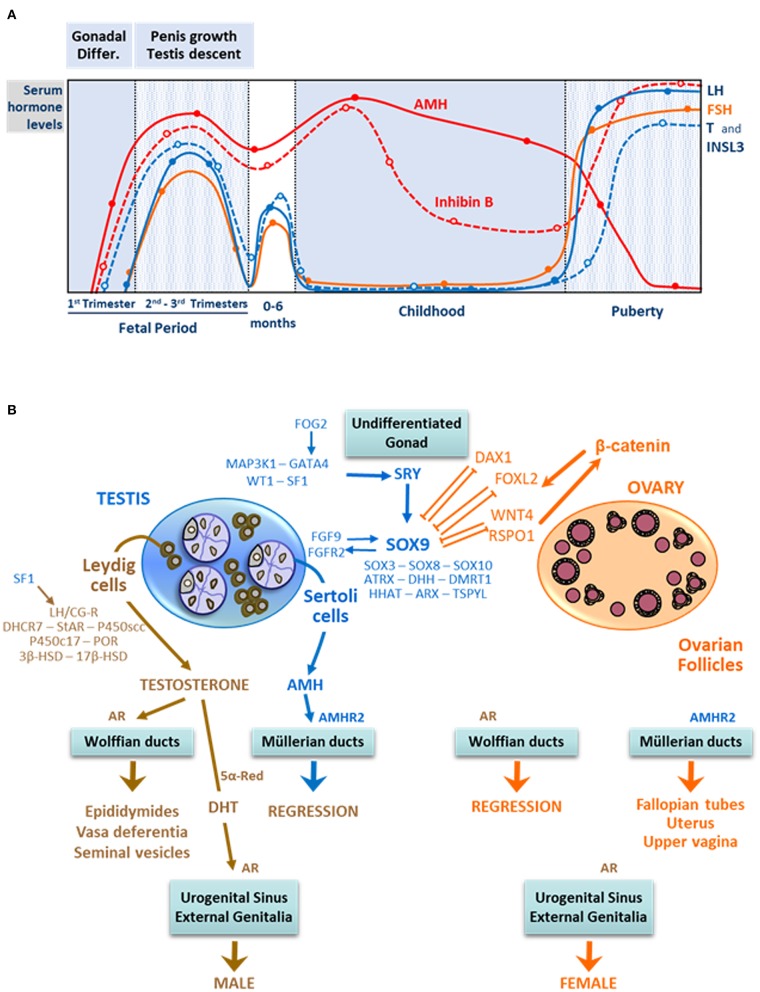
Male reproductive hormone levels and fetal sex differentiation. **(A)** Ontogeny of male reproductive hormone levels from fetal to adult periods of life. In the early fetal stage, the initiation of testicular hormones secretion is independent of luteinizing hormone (LH) and follicle-stimulating hormone (FSH). In the second and third trimesters, LH and FSH are the main regulators of Sertoli and Leydig cell hormone production. For ~6 months after birth, LH, FSH, and testicular hormone levels remain at high levels. Afterwards, in infancy and childhood, gonadotropins, testosterone (T), and insulin-like factor 3 (INSL3) levels decrease, while those of anti- Müllerian hormone (AMH) and inhibin B continue to be high. During puberty, gonadotropins, T and INSL3 increase again to attain adult levels. AMH is inhibited by testosterone, and inhibin B is stimulated by FSH. Reprinted, with permission, from Salonia et al. (4). © 2019 Springer Nature Limited. **(B)** The undifferentiated gonad is exposed to pro-testicular and pro-ovarian factors. In the XY fetus, SRY expression regulated by MAP3K1, GATA4, SF1, and WT1 shifts the balance toward the pro-testicular factors, like SOX9, inducing testis differentiation. Sertoli cells produce anti-Müllerian hormone (AMH), which provokes Müllerian duct regression upon binding to its receptor AMHR2. Leydig cells express the LHCG receptor and steroidogenic proteins involved in androgen synthesis; testosterone acts on Wolffian ducts, the urogenital sinus, and the external genitalia, either directly or after metabolization to the more potent androgen dihydrotestosterone (DHT), to virilize them. In the XX fetus, where SRY is absent, the pro-ovarian factors WNT4, RSPO1, FOXL2, and DAX1 control the differentiation of the ovaries. The latter do not produce androgens or AMH, which results in feminization of the internal and external genitalia. Modified with permission from Rey et al. (13) © 2016 Elsevier Saunders.

Gonadal differentiation into ovaries or testes, in the 7th week of embryonic life, depends on a complex network of genes: the presence of the *SRY* gene on the Y chromosome disrupts the balance between pro-testicular and pro-ovarian genes, triggering testis differentiation (9–12). Subsequently, the action of two discrete testicular hormones, AMH and androgens, drive the differentiation of the internal ducts, urogenital sinus, and external genitalia (Figure 2). AMH, secreted by Sertoli cells, provokes the regression of Müllerian ducts in fetal weeks 8 and 9 (14). Androgens produced by Leydig cells induce the stabilization and differentiation of Wolffian ducts as well as the virilization of the urogenital sinus and the external genitalia between fetal weeks 8 and 13. These processes occurring in the first trimester of fetal life are independent of fetal pituitary gonadotropins: basal AMH expression is driven by a set of transcription factors (15), and androgen production is regulated by placental hCG (16). Insulin-like factor 3 (INSL3), produced by Ledyig cells, is involved in testicular descent (17). Ovarian hormones have no influence on fetal sex differentiation (18).

In the newborn, testosterone, INSL3, AMH, and inhibin B are significantly higher in the male than in the female. During childhood, testosterone and INSL3 levels are similar between males and females, but AMH and inhibin B are clearly different between sexes, with higher levels in males (19–21).

### DSD: Abnormal Fetal Sex Differentiation

The process of fetal sex differentiation can be disrupted at any of the three stages. The early morphogenesis of the reproductive tract may fail independently of gonadal differentiation: these are malformative, non-endocrine DSD (22) and will not be dealt with in this review. Gonadal differentiation failure is known as gonadal dysgenesis and results in primary hypogonadism. Testicular dysgenesis drives to incomplete virilization, and the condition is known as dysgenetic DSD (11, 14, 23). According to the gene defect and probably the timing of its abnormal expression, variable degrees of dysgenesis will result, thus eliciting variable compromise of endocrine and paracrine secretion of androgens and AMH. This will be reflected in different degrees of virilization of the external genitalia and Wolffian ducts as well as in the magnitude of regression of Müllerian structures. Conversely, ovarian dysgenesis does not affect the development of female genitalia. Finally, the third stage of fetal sex differentiation can be disrupted either by a deficient production of testicular hormones (24, 25) or an impaired action due to receptor defects (26, 27) in the XY fetus, or to an excess androgen exposure in the XX fetus (28–30). In this review, we will address impaired testicular endocrine function during fetal life resulting in incomplete virilization.

### Hypogonadism: Classifications

Male hypogonadism, i.e., the impaired function of the testes as compared to what is expected for age, may involve a decreased hormone secretion (AMH, inhibins, and/or androgens) and/or an impaired sperm production. It can be due to a primary disorder or secondary to a defect in the hypothalamic-pituitary axis, respectively resulting in primary (testicular or hypergonadotropic) or secondary (central or hypogonadotropic) hypogonadism (Table 1). The terms hypergonadotropic and hypogonadotropic, extensively used in adult medicine (4), should be applied with caution in pediatric patients (6). Indeed, although elevated gonadotropins, especially FSH, are a typical feature of primary hypogonadism in newborns and infants (31), normal gonadotropin levels can occur in up to 30–70% of anorchid boys later in childhood (32). This highlights that, throughout childhood, the normal development of the intrinsic central inhibitory tone exerted on GnRH pulse generator can overcome the rise of FSH due to low serum inhibin B in circulation.

**Table 1:** Classification of fetal-onset male hypogonadism.

	**Primary Hypogonadism**	
	**Whole gonadal dysfunction**	**Dissociated gonadal dysfunction**
**First trimester**	Gonadal dysgenesis	Leydig cells: LHCG-R mutation, steroidogenic defects
		Sertoli cells: AMH mutation
	**Central Hypogonadism**	
	**Whole gonadal dysfunction**	**Cell-specific gonadal dysfunction**
**Second–third trimesters**	Multiple pituitary hormone deficiency	Leydig cells: LHβ-subunit gene mutation
	Isolated hypogonadotropic hypogonadism (IHH)	Sertoli cells: FSHβ-subunit gene mutation

As regards to the testicular compartment that is primarily affected, hypogonadism may present with whole gonadal dysfunction or a dissociated, cell-specific gonadal dysfunction. In the latter, one of the cell populations (Sertoli, Leydig, or germ cells) is primarily disrupted while the function of the others remains intact, at least for some time (5). Gonadal dysgenesis is an example of primary hypogonadism with whole gonadal dysfunction. Mutations of the FSH receptor and AMH genes are examples of Sertoli cell-specific hypogonadism. Mutations of the LH receptor and of steroidogenic proteins provoke Leydig cell-specific hypogonadism. Finally, deletions of the Y chromosome leading to azoospermia are examples of germ cell-specific hypogonadism.

Finally, hypogonadism may be congenital or acquired during postnatal life. The clinical presentation varies according to the age at which hypogonadism is established (4, 33, 34); particularly, fetal-onset hypogonadism results in DSD only when it is established in the first trimester of gestation, as it can be deduced from the knowledge of the physiology of fetal sex differentiation discussed above.

## Hypogonadism and Endocrine-Related DSD

Usually, the initial diagnostic approach of patients with DSD is based on the karyotype (35). In 46,XY individuals, the etiology of DSD is classified into: disorders of gonadal development (or gonadal dysgenesis), disorders of androgen synthesis (in non-dysgenetic gonads), disorders of androgen action, and disorders of AMH synthesis or action (resulting in the Persistent Müllerian Duct Syndrome, PMDS) (36). Gonadal dysgenesis and isolated disorders of androgen or AMH synthesis represent early fetal-onset forms of primary hypogonadism resulting in 46,XY DSD (31). Disorders of androgen or AMH action do not initially affect testicular hormone production and, together with non-endocrine DSD (or unclassified disorders), will not be further discussed in this review.

Disorders of gonadal development (or gonadal dysgenesis) also occur in sex-chromosomal DSD (35). The most frequent forms of sex-chromosome aneuploidies, Turner (45,X) and Klinefelter (47,XXY) syndromes, generally do not present at birth with genital anomalies. Therefore, they do not appear as a differential diagnosis in the newborn. Conversely, patients with 45,X/46,XY or 46,XX/46,XY (or other mosaicisms/chimerisms) frequently bear diverse forms of gonadal dysgenesis (partial testicular dysgenesis, asymmetric gonadal differentiation, ovotestes, etc.) which lead to the existence of ambiguous genitalia. Furthermore, ovotestes and dysgenetic testes can develop in 46,XX fetuses, both in the presence or absence of *SRY* (37).

### Disorders of Gonadal Development: Early Fetal-Onset Primary Hypogonadism With Whole Gonadal Dysfunction

Regardless of the karyotype and the pathogenesis, gonadal dysgenesis represents a typical form of primary hypogonadism established in the first trimester of fetal life. Because this is the period when internal and external genitalia are masculinized in the presence of testicular hormones or feminized in their absence, primary hypogonadism results in deficient masculinization of the fetus carrying a Y chromosome (46,XY or chimeras or mosaicisms with a Y chromosome) or in XX fetuses carrying a translocated *SRY* or gene imbalances provoking ovotesticular or dysgenetic testicular development, as previously explained.

**Figure 3:**
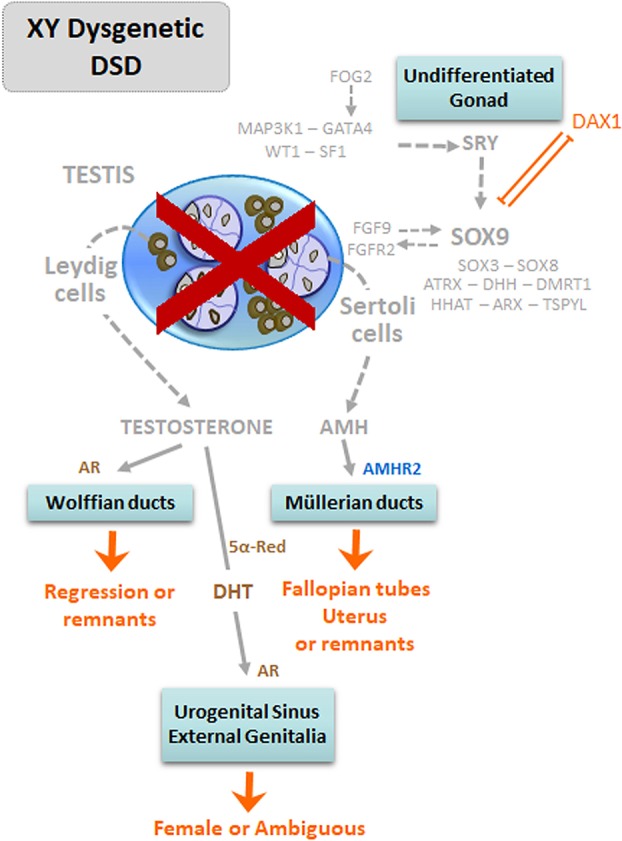
Dysgenetic DSD in 46,XY patients: early fetal-onset primary hypogonadism with whole gonadal dysfunction. The lack of normal testis differentiation, due to mutations of pro-testicular genes or to overexpression of DAX1 results in gonadal dysgenesis. Consequently, the insufficient production of androgens and anti-Müllerian hormone (AMH) results in undervirilization or complete feminization of the internal and external genitalia. Modified with permission from Rey et al. (13) © 2016 Elsevier Saunders.

The impaired gonadal development is reflected in a dysfunction of all testicular cell populations: Sertoli cells are less numerous, thus resulting in an insufficient AMH output leading to the existence of Müllerian remnants (Figure 3). Germ cells are also scarce and less differentiated, with a consequently increased risk of infertility and testicular neoplasia (38, 39). Leydig cell number and function is also impaired, resulting in a hypoandrogenism that leads to undervirilization of the internal and external genitalia.

**Figure 4:**
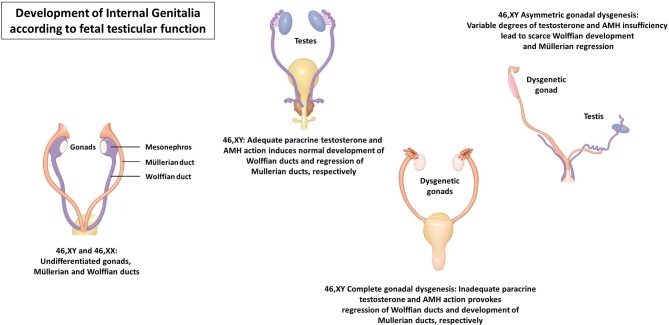
Development of the internal genitalia according to fetal testicular function.

The most severe forms of dysgenesis—pure or complete gonadal dysgenesis—present with a complete development of the uterus and Fallopian tubes, reflecting AMH deficiency, and absence of Wolffian remnants together with female external genitalia, as a consequence of androgen deficiency (Figure 4). In partial forms of gonadal dysgenesis, the degree of virilization is variable depending on the mass of functional testicular tissue: from micropenis, hypospadias, and cryptorchidism with variable development of Wolffian and Müllerian remnants to an almost female phenotype in severe cases (40). When the condition is asymmetric—known as mixed gonadal dysgenesis (41) or asymmetric gonadal differentiation (42)—, a hemi-uterus and a fallopian tube exist on the side of the streak gonad, and external genitalia may also show asymmetric development.

#### Gonadal Dysgenesis in 46,XY patients

Gonadal dysgenesis may be isolated or associated with dysmorphic features of non-reproductive organs (Table 2). Isolated gonadal dysgenesis is caused by mutations in pro-testicular genes. Inactivating mutations or deletions of *SRY* are present in ~15% of 46,XY females with pure gonadal dysgenesis (Swyer syndrome) (43, 44). Non-reproductive organs are not affected. Loss-of-function variants of *MAP3K1* lead to an increased expression of pro-ovarian factors, like β-catenin, and reduced expression of pro-testicular factors, like SOX9/FGF9 (45). A similar pathogenesis has been proposed for mutations in *ZNRF3* (46). Dysgenetic DSD can also be attributed to mutations in *DHX37* (47, 48) particularly those individuals exhibiting the embryonic testicular regression syndrome and rearrangements involving *SOX8* (49). Finally, isolated gonadal dysgenesis was described in 46,XY females with partial duplications of Xp21.3–p21.2, known as the DSS (dosage sensitive sex-reversal) locus encompassing *NR0B1* that encodes DAX1; the increased expression of DAX1 is believed to impair testis differentiation (50, 51).

**Table 2:** Disorders of Sex Development (DSD) due to 46,XY gonadal dysgenesis.

**Associated syndrome**	**Implicated gene**	**Locus**
Swyer syndrome or partial testicular dysgenesis	*SRY*	Yp11.2
	*MAP3K1*	5q11.2
	*ZNRF3*	22q12.1
	*DSS*	Xp21.2
	*DHX37*	12q24.31
Campomelic dysplasia: bowing of long bones, hip dislocation, scapula hypoplasia, small thoracic cage, micrognathia, macrocephaly, low-set ears, flat nasal bridge, and cardiac and renal defects	*SOX9*	17q24.3
Denys-Drash syndrome: nephropathy and Wilms tumor	*WT1*	11p13
Frasier syndrome: nephropathy and gonadoblastoma		
Adrenal insufficiency	*NR5A1*	9q33.3
ATRX syndrome: α-thalassemia and mental retardation	*ATRX*	Xq21.1
Minifascicular neuropathy	*DHH*	12q13.12
Clinodactyly, hydrocephaly, and autistic spectrum disorder	*FOG2/ZFPM2*	8q23.1
Craniosynostosis	*FGFR2*	10q26.13
Microcephaly, mental retardation, short stature, and/or bronchial or digestive malformations	*DMRT1/DMRT2*	9p24.3
Chondrodysplasia, cerebellar hypoplasia, and coloboma of the optic disks	*HHAT*	1q32.2
Lissencephaly and epilepsy	*ARX*	Xp21.3
Cardiac malformations	*MYRF*	11q12.2
Sudden death	*TSPYL*	6q22.1

Gonadal dysgenesis associated with dysmorphic features of non-reproductive organs (Table 2) has been seen in patients with mutations or deletions of *SOX9* (52), *WT1* (53), *NR5A1* encoding SF1 (54), *ATRX* (55), *DHH* (56), *FOG2*/*ZFPM2* (57), *FGFR2* (58), 9p24 encompassing *DMRT1* and *DMRT2* (59), *HHAT* (60), *ARX* (61), *MYRF* (62), and *TSPYL* (63). Mutations in other genes, like *CBX2* (64), *ESR2* (65), *MALD1* (66, 67), *STARD8* (68), and *WWOX* (69), have been described in patients with gonadal dysgenesis but their pathogenic relationship still needs to be clearly established.

In a newborn with complete (or pure) gonadal dysgenesis, the external genitalia are typically female. There is also a normal development of the Fallopian tubes and uterus. The phenotype is due to a complete lack of androgen and AMH secretion. Because of the typical female aspect, the diagnosis is not suspected until pubertal age. Then, the absence of pubertal signs is the main complaint. Gonadal hormones are undetectable (14) and gonadotropins are very high in newborns. During childhood, gonadotropin levels decrease to almost normal values by the age of 6–9 years (70), but they increase again to extremely high levels in pubertal age. Replacement therapy with estrogens and progestins results in normal development of secondary sex characteristics, and gestations have been successful after oocyte donation (71). Gonads are characterized by the existence of bilateral fibrous streaks, with no gonadal tissue, although in rare cases abnormal germ cells can be observed embedded in cord-like structures. The risk of gonadoblastoma after puberty is increased.

In patients with partial dysgenesis, the degree of virilization of the internal and external genitalia correlates with the amount of functional testicular tissue present during the first trimester of intrauterine development exerting endocrine and paracrine hormonal action. The mildest forms may present as males with infertility. Serum levels of AMH and androgens are low for males but above the female range, while gonadotropins are elevated, though less than in pure gonadal dysgenesis. Although low INSL3 production might be involved in failure of testicular descent, no information is available on serum INSL3 in patients with dysgenetic DSD. Testes are small, have a thin albuginea with scarce seminiferous tubules separated by wide spaces of fibrous connective tissue. Germ cell number is significantly decreased, and gonadal tumor risk is increased (38, 72, 73). The trend for male assignment in these cases has increased over the last decades (74), and the surgical repair of hypospadias and management of tumor risk have become the main challenges in the management (36). At the age of puberty, replacement with testosterone leads to satisfactory development of secondary sex characteristics and growth, but infertility is the rule in adulthood (71).

#### Gonadal Dysgenesis in 46,XX Patients

As already mentioned, ovarian dysgenesis has no impact on fetal differentiation of the female genitalia; these patients do not seek medical assistance until the age of puberty, when the lack of ovarian steroids results in delayed puberty. These disorders will not be discussed amongst the DSD due to fetal hypogonadism addressed in this review. Congenital adrenal hyperplasia, aromatase deficiency, and other conditions characterized by fetal virilization not due to fetal-onset hypogonadism are also out of the scope of this review.

Disorders of gonadal differentiation leading to the development of testicular tissue and ambiguous or male genitalia may occur in 46,XX individuals. In the originally described “XX male” (75), testes differentiate almost normally—only minor signs of dysgenesis are present, characterized by lower numbers of germ cells (76)—and the patients are fully virilized. These patients usually seek medical attention in adulthood owing to infertility (34). This is a typical case of a cell-specific dissociated hypogonadism, where only the germ cell population is initially affected: spermatogenesis is disturbed by the presence of two X chromosomes and the lack of Y-chromosome genes that are essential for germ cell development. Approximately 90% of XX males are *SRY*-positive, i.e., they bear part of the short arm of the Y chromosome translocated onto the X chromosome or an autosome (77).

*SRY*-negative cases have been intriguing for decades (78), until the importance of a balance between pro-testicular and pro-ovarian genes was apprehended. The development of testicular tissue has been attributed to: (i) overexpression of pro-testicular genes, (ii) insufficient expression of pro-ovarian genes, and (iii) mixed or unknown pathogenic mechanisms (37).

Overexpression of testicular factors was initially described in patients bearing duplications of the *SOX9* gene or duplications/triplications of its regulatory sequences (79–85), and subsequently found in individuals with duplications or rearrangements of the *SOX3* gene (84, 86–88) or its regulatory sequences (89, 90), and duplications of *SOX10* (91–95) or *FGF9* (96). Insufficient expression of pro-ovarian genes has been proposed as the underlying pathogenesis in patients with loss-of-function mutations or deletions of *RSPO1* (97–100) or *WNT4* (101). More recently, 46,XX testicular or ovotesticular DSD has been attributed to mutations in *NR5A1*, encoding SF1 (102–107), *WT1* (108) and *NR2F2*, encoding COUP-TF2 (109, 110).

The resulting phenotype can be either testicular or ovotesticular DSD; the testicular tissue usually shows signs of typical dysgenesis, justifying a primary hypogonadism with whole gonadal dysfunction. External genitalia are ambiguous, reflecting insufficient androgen levels to fully virilize the fetus, and Müllerian remnants may be present, indicating deficient AMH production.

Sex assignment in these cases follows the general suggestions for DSD (2). In patients raised as boys, there is a need for ovarian tissue removal, if present, before the age of puberty to prevent potential complications of cystic follicle development and to avoid gynecomastia due to estrogen secretion (111). The risk of tumor development in the testicular tissue seems to be low, likely due to the absence of Y-chromosome peri-centromeric sequences (72). In patients with ambiguous genitalia and a diagnosis of ovotesticular DSD, attention should be driven to the fact that the ovarian tissue may produce oocytes after pubertal development, which raises the possibility of fertility (112). In these cases, assignment to the female gender may be preferred, and special care should be taken during testicular tissue removal to preserve the ovarian tissue.

#### Gonadal Dysgenesis in Sex-Chromosome DSD

The lack of the short arm of the Y chromosome, where *SRY* maps, is responsible for gonadal dysgenesis in patients with sex-chromosome mosaicisms or chimeras. It is noteworthy that the frequent sex-chromosome aneuploidies observed in patients with Klinefelter syndrome (47,XXY and variants) and in triple X syndrome (47,XXX and variants) most usually present typical genitalia at birth and do not represent a differential diagnosis of the conditions discussed in this review. Several different karyotypes have been described in patients with sex-chromosome DSD presenting with ambiguous genitalia. The most prevalent is 45,X/46,XY, which is usually—though not always—associated with asymmetric gonadal differentiation (41, 42, 73). 46,XX/46,XY chimeras usually present with ovotesticular DSD.

### Disorders of Testicular Hormone Synthesis: Early Fetal-Onset Primary Hypogonadism With Dissociated Dysfunction

Congenital specific disorders of androgen or AMH synthesis are examples of non-dysgenetic, early-onset, primary hypogonadism characterized by a cell-specific testicular dysfunction in 46,XY patients. The main feature is that only Leydig cell function is impaired in one case and exclusively Sertoli cell secretion is affected in the other, leading to specific clinical and biochemical presentations.

#### 46,XY DSD Due to Disorders of Androgen Synthesis

**Figure 5:**
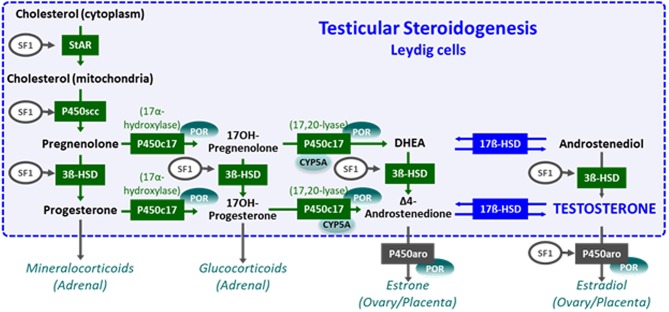
Steroidogenesis. The steroidogenic pathway occurring in testicular Leydig cells is schematically shown in the box. Steroidogenic steps taking place in the adrenals, placenta, and ovaries are outside the box. Modified with permission from Rey et al. (14) © 2010 Elsevier Ltd.

**Figure 6:**
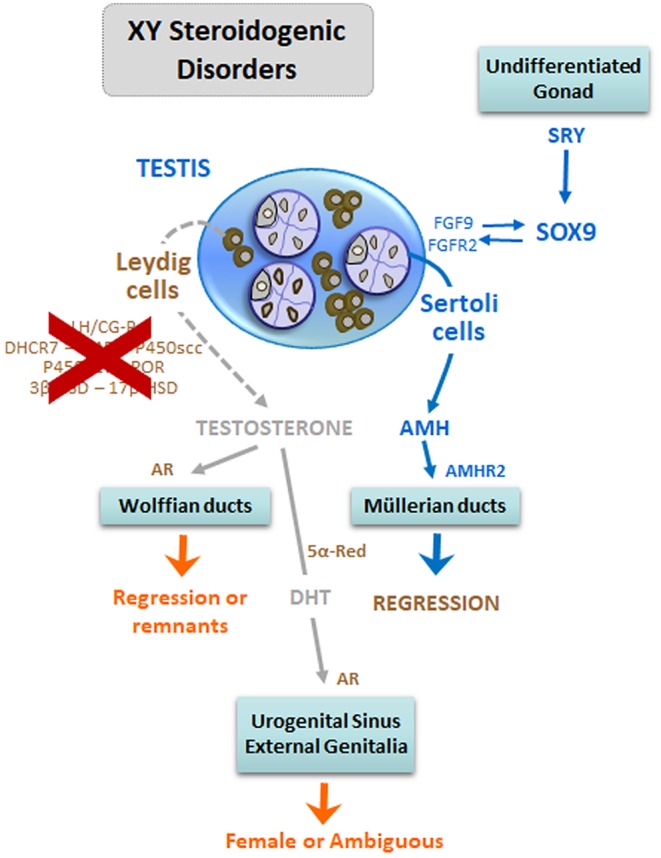
DSD due to steroidogenic defects in 46,XY patients: early fetal-onset primary hypogonadism with Leydig cell-specific dysfunction. The lack of Leydig cell differentiation, due to mutations in the LHCGR gene, or in steroidogenic proteins leads to insufficient androgen production by the testes resulting in undervirilization or complete feminization of the external genitalia. In these patients, there is no uterus or Fallopian tubes because anti-Müllerian hormone (AMH) is produced by Sertoli cells. Modified with permission from Rey et al. (13) © 2016 Elsevier Saunders.

The main sources of androgens are the gonads and the adrenals. There are steps of steroidogenesis that are common to the testis and the adrenal cortex, and others are specific to the gonads (Figure 5) (24, 29). Disorders of androgen synthesis represent a “dissociated” or “cell-specific” fetal-onset primary hypogonadism, characterized by impaired Leydig cell, but normal Sertoli cell, function. Testosterone levels are low, but serum AMH is within the normal male range or elevated (113), and Müllerian derivatives are absent (Figure 6). Steroidogenic defects, like that of insufficient testosterone conversion to dihydrotestosterone (DHT) occurring locally in the end-organ due to mutations in the gene encoding 5α-reductase type 2, will not be discussed in this review, since they do not represent a form of impaired gonadal function.

##### Disorders of androgen synthesis affecting the testes and the adrenals

Cholesterol is the initial substrate for steroid synthesis. Smith-Lemli-Opitz Syndrome (SLOS) results from a defect in dehydrocholesterol reductase (DHCR), involved in the last step of cholesterol synthesis. Cholesterol also plays a major role in the formation of the nervous system, face, and limbs; therefore, patients with SLOS present with DSD associated with polymalformations including microcephaly, facial malformations, growth retardation and congenital heart defects (114). Clinical diagnosis of SLOS is suspected in patients with elevated 7–dehydrocholesterol and low cholesterol.

The first step of gonadal and adrenal steroidogenesis is cholesterol transfer from the cytoplasm into the inner mitochondrial membrane (Figure 5), under control of the steroidogenic acute regulatory protein (StAR). Defects in the *STAR* gene lead to lipoid congenital adrenal hyperplasia. Most 46,XY newborns present with female external genitalia and severe glucocorticoid and mineralocorticoid deficiency. Milder forms have been reported with ambiguous or male genitalia. All steroids are low and no response is observed after hCG and ACTH stimulation. There is massive adrenal enlargement, due to accumulation of cholesterol esters in the adrenal cortex (115).

Subsequently, cytochrome P450 cholesterol side-chain cleavage enzyme cytochrome P450 (P450scc) catalyzes the synthesis of pregnenolone. P450scc deficiency, due to mutations in *CYP11A1*, also results in very low steroid levels but without adrenal enlargement. Most 46,XY patients are born with female external genitalia, but partial deficiencies present with ambiguous genitalia (115, 116).

Cytochrome P450c17 has two enzymatic activities (Figure 5): 17α-hydroxylase, which hydroxylates pregnenolone or progesterone into 17α-hydroxypregnenolone or 17α-hydroxyprogesterone (17-OHP), and 17,20-lyase, required for the synthesis of dehydroepiandrosterone (DHEA) and Δ4-androstenedione. Mutations in *CYP17A1*, encoding P450c17, may induce a combined deficiency of 17α-hydroxylase and 17,20-lyase, but isolated 17,20-lyase deficiency has also been described. The 46,XY newborns are undervirilized. Progesterone and 17-OHP are high in serum whereas DHEA, Δ4-androstenedione, and testosterone are low. Combined 17α-hydroxylase/17,20-lyase deficiency leads to hypertension and hypokalemia due to the accumulation of deoxycorticosterone and corticosterone. In isolated 17,20-lyase deficiency, testicular, but not adrenal, function is impaired (116, 117).

Cytochrome b5 enhances 17,20-lyase activity of P450c17 but does not modify 17-hydroxylase activity. It also reduces methemoglobin (ferric hemoglobin) to normal hemoglobin (ferrous hemoglobin). Mutations in *CYB5A* result in 46,XY DSD with impaired 17,20-lyase activity and methemoglobinemia (116).

P450 oxidoreductase (POR) is essential for the catalytic activity of P450c17 (preferentially its 17,20-lyase activity), 21-hydroxylase and aromatase (116, 118). XY newborns with *POR* mutations are undervirilized but present mild signs of congenital adrenal hyperplasia (e.g., elevated 17-OHP), and their mothers virilize due to impaired placental aromatase. Severe mutations lead to the Antley-Bixler syndrome, characterized by craniosynostosis, fusion of long bones, midface hypoplasia, and choanal stenosis. Laboratory findings reflect the combined deficiencies of 17α-hydroxylase and 21-hydroxylase: elevated serum progesterone and 17-OHP but low DHEA and androstenedione.

3β-hydroxysteroid dehydrogenase (HSD) type 2 catalyzes the conversion of Δ5- to Δ4-steroids (pregnenolone to progesterone, 17α-hydroxypregnenolone to 17-OHP, DHEA to androstenedione, and androstenediol to testosterone; Figure 5). Mutations in *HSD3B2* leads to 3β-HSD type 2 deficiency, characterized by ambiguous genitalia and variable adrenal impairment (116). Laboratory findings are characterized by an elevated Δ5/Δ4 steroids ratio, particularly after ACTH or hCG stimulation.

##### Disorders of androgen synthesis specifically affecting the testes

LH and hCG bind to the same receptor on the Leydig cell membrane to induce their typical steroidogenic phenotype. Inactivating mutations in the *LHCGR* gene lead to Leydig cell aplasia or hypoplasia resulting in various degrees of undervirilization: from micropenis, hypospadias, and cryptorchidism to female external genitalia. All androgen levels are low in basal conditions and after hCG stimulation, but adrenal steroid response to ACTH is normal (119).

The enzyme 17β-HSD type 3 converts Δ4-androstenedione to testosterone in the testes (Figure 5) (24, 116). *HSD17B3* gene mutations lead to 46,XY DSD characterized by a female or ambiguous external genital phenotype and unaffected adrenal function. Virilization may occur at pubertal age owing to androgen synthesis by other isoforms, like that encoded by *HSD17B5* (also called *AKR1C3*) involved in the “backdoor” steroidogenic pathway (116). Laboratory findings are characterized by elevated Δ4-androstenedione and low testosterone after hCG stimulation (24).

#### 46,XY DSD Due to Disorders of AMH Synthesis

**Figure 7:**
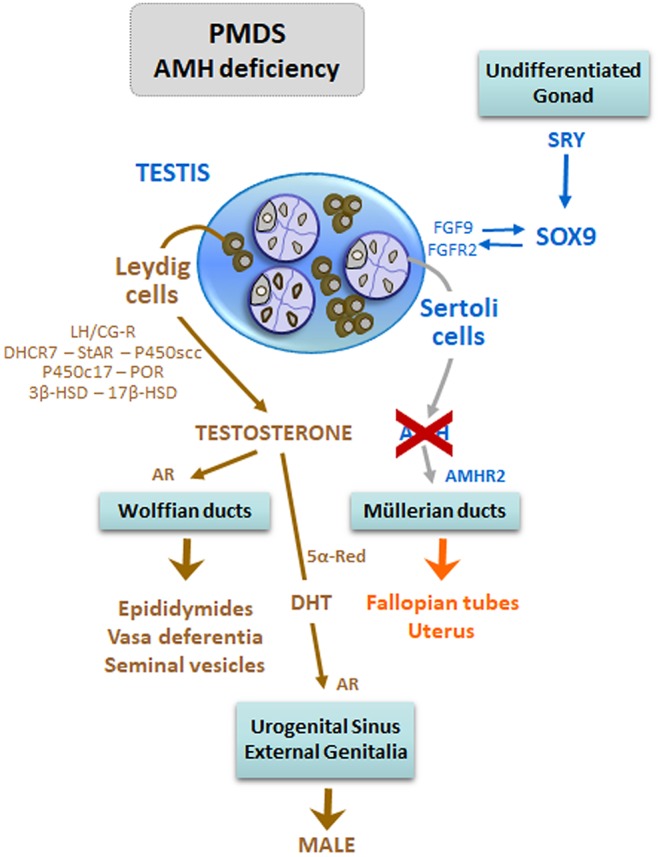
DSD due to anti-Müllerian hormone (AMH) deficiency in 46,XY patients: early fetal-onset primary hypogonadism with Sertoli cell-specific dysfunction. Mutations of the *AMH* gene are responsible for the persistence of the uterus and Fallopian tubes, a condition known as Persistent Müllerian Duct Syndrome (PMDS), in otherwise normally virilized newborns. Modified with permission from Rey et al. (13) © 2016 Elsevier Saunders.

Defects in Sertoli cell AMH secretion, due to mutations in the *AMH* gene, are a typical form of “dissociated” or “cell-specific” fetal-onset primary hypogonadism, characterized by impaired Sertoli cell, but normal Leydig cell, function (Figure 7). The resulting disorder is known as the persistent Müllerian duct syndrome (PMDS), which is characterized by the presence of the uterus and Fallopian tubes in an otherwise normally virilized newborn (27). The existence of ambiguous external genitalia, which reflects a concomitant defect in androgen synthesis, rules out the diagnosis of PMDS. Clinical manifestations are cryptorchidism with or without inguinal hernia, and the diagnosis is not suspected until Müllerian derivatives are unexpectedly found at surgery. Mutations in the *AMH* gene explain ~45% of PMDS patients. Serum testosterone and gonadotropins are in the male range. AMH is undetectable in patients with *AMH* mutations. In patients with PMDS due to AMH receptor defects, testicular function is not affected and AMH levels are within the male range (25). Malignant tumors of the testes occur in ~1/3 of adults with the disorder, while malignancies of the Müllerian derivatives are less frequent (27).

## Early Fetal-Onset Central Hypogonadism Does Not Lead to DSD

The GnRH neurons derive from cells present in the nasal placode (120), which migrate together with olfactory axons through the cribriform plate and arrive in the developing forebrain in the 9th−10th weeks. The pituitary develops from the Rathke's pouch, and functional gonadotropes secrete LH from fetal week 12 (121), when sex differentiation is almost complete. As mentioned, Leydig cells secrete androgens driven by hCG rather by pituitary LH during the period of sex differentiation. This explains why 46,XY fetuses virilize in spite of complete absence of gonadotropin secretion in congenital central (hypogonadotropic) hypogonadism. Because testicular androgen synthesis is dependent on fetal LH during the second and third trimesters, these patients present with micropenis and cryptorchidism at birth (31).

## Concluding Remarks

**Figure 8:**
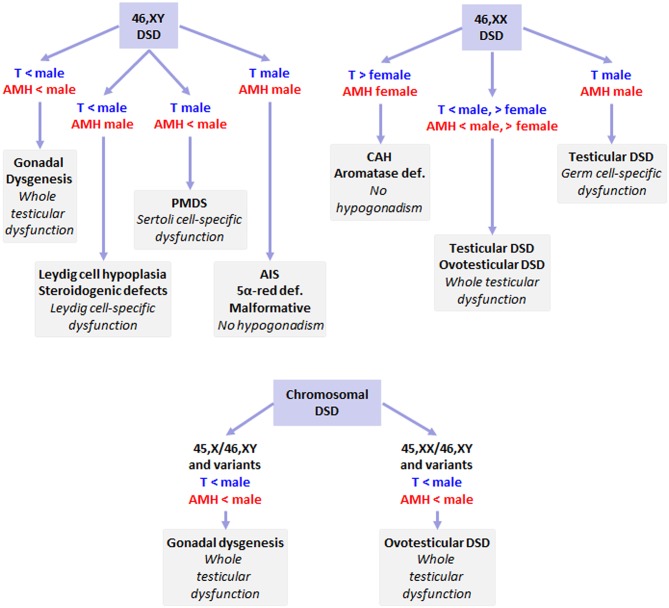
DSD and fetal-onset male hypogonadism. 46,XY DSD may result from various forms of primary fetal-onset male hypogonadism, or from defects in hormone action in target organs (AIS: androgen insensitivity syndrome, or 5α-reductase deficiency), without hypogonadism. In 46,XX individuals with testicular differentiation, partial virilization results from impaired testicular function; in completely virilized newborns, Leydig and Sertoli cell function is preserved and only germ cell development is impaired; however, the most frequent situation of XX virilization results from dysfunction of the adrenals (e.g., congenital adrenal hyperplasia, CAH) or the placenta (aromatase deficiency). Chromosomal DSD with 45,X/46,XY or 46,XX/46,XY karyotypes, testicular tissue is characterized by impaired function of all cell populations, as shown by the existence of testosterone and AMH levels that are below the male range. AIS, androgen insensitivity syndrome; CAH, congenital adrenal hyperplasia; Def., deficiency; Malformative, anatomical malformation of the genitalia not due to hormonal disorders; PMDS, persistent Müllerian duct syndrome, due to specific AMH defects, < or > female or male: below or above normal female or male range.

In patients with a Y chromosome (46,XY, 45,X/46,XY, or variants), DSD usually results from testicular dysgenesis, an early fetal-onset hypogonadism characterized by whole gonadal dysfunction presenting with serum levels of testosterone and AMH that are below the male range (Figure 8). In 46,XY individuals, DSD may result from isolated Leydig cell dysfunction; this dissociated form of fetal-onset hypogonadism is characterized by androgen levels below the male range and AMH within the male range. When there is an isolated Sertoli cell dysfunction, AMH is low while testosterone is within the male range. If both testosterone and AMH are within the male range, DSD is not due to fetal-onset hypogonadism, but rather to a defect in the target organ, e.g., androgen insensitivity or 5α-reductase deficiency, or to an anatomical malformation. In 46,XX newborns with ambiguous or male genitalia, testicular or ovotesticular DSD may be the underlying cause; Sertoli and Leydig cell populations may be affected, resulting in undervirilization but they may be functionally normal, like in the XX male presenting with testosterone and AMH levels within the male range. In newborns with androgen levels above but AMH within the female range, virilization is not related to hypogonadal states but rather to adrenal or placental dysfunction. In chimeric forms of DSD, e.g., 46,XX/46,XY or variants, ovotesticular differentiation usually occurs, characterized by androgen and AMH levels that are above the female but below the male range, indicating that there is a whole testicular tissue dysfunction. All these are primary forms of fetal hypogonadism. Fetal-onset central hypogonadism usually leads to micropenis or cryptorchidism, reflecting insufficient androgen production in the second half of intrauterine life, but does not result in ambiguous genitalia.

## Author Contributions

All authors listed have made a substantial, direct and intellectual contribution to the work, and approved it for publication.

### Conflict of Interest

The authors declare that the research was conducted in the absence of any commercial or financial relationships that could be construed as a potential conflict of interest.
